# Discriminative Capacity of Visceral Adiposity and Triglyceride Glucose-Waist Circumference Indices for Metabolic Syndrome in Spanish Adolescents: A Cross-Sectional Study

**DOI:** 10.3390/metabo15080535

**Published:** 2025-08-07

**Authors:** Ángel Fernández-Aparicio, Miriam Mohatar-Barba, Javier S. Perona, Jacqueline Schmidt-RioValle, Carmen Flores Navarro-Pérez, Emilio González-Jiménez

**Affiliations:** 1 Department of Nursing, Faculty of Health Sciences, University of Granada, 18016 Granada, Spain; anfeapa@ugr.es (Á.F.-A.); jschmidt@ugr.es (J.S.-R.); emigoji@ugr.es (E.G.-J.); 2 Instituto de Investigación Biosanitaria (ibs.GRANADA), 18016 Granada, Spain; miriamb@ugr.es; 3 Department of Nursing, Faculty of Health Sciences, Melilla Campus, University of Granada, 52005 Melilla, Spain; 4 Department of Food and Health, Instituto de la Grasa-CSIC, Campus of the University Pablo de Olavide, 41013 Seville, Spain; 5 Department of Nursing, Faculty of Nursing, Physiotherapy and Podiatry, University of Seville, 41009 Seville, Spain; cnperez@us.es

**Keywords:** anthropometric indices, early detection, metabolic health, nutrition-related biomarkers, obesity, preventive nutrition

## Abstract

**Background/Objectives:** Adolescence is a critical period for the early detection of metabolic syndrome (MetS), a condition that increases the risk of cardiometabolic diseases in adulthood. Timely identification of at-risk adolescents enables targeted prevention strategies. This study aimed to analyze the discriminative capacity and accuracy of six biochemical and/or anthropometric indices related to lipid metabolism and adiposity for the early detection of MetS in a sample of Spanish adolescents. **Methods:** A cross-sectional study carried out according to the STROBE guidelines. A sample of 981 adolescents aged 11–16 years old were randomly recruited from schools in Southeastern Spain. The presence or absence of MetS was determined according to the International Diabetes Federation criteria. The following biochemical and/or anthropometric indices were evaluated: triglyceride glucose index, visceral adiposity index, logarithm children’s lipid accumulation product, triglyceride glucose-body mass index, triglyceride glucose-waist circumference, and triglyceride glucose-waist-to-hip ratio. **Results:** The triglyceride glucose-waist-to-hip ratio and triglyceride glucose-body mass index parameters were the strongest indicators associated with MetS in boys and girls, respectively, after adjusting for several factors. Moreover, all evaluated indices showed optimal AUC values, with the visceral adiposity index and triglyceride glucose-waist circumference index exhibiting the highest discriminative capacity in both genders. **Conclusions:** The evaluated biochemical and anthropometric indices—particularly visceral adiposity index and triglyceride-glucose-waist circumference—show promise as accessible biomarkers for identifying adolescents at metabolic risk. These indices may serve as practical tools in preventive health strategies aimed at improving metabolic health by screening adolescents at risk of MetS, thereby helping to reduce the future burden of non-communicable diseases.

## 1. Introduction

Non-communicable diseases are responsible for five out of ten health problems worldwide, including cardiovascular disease, the development of which is closely linked to the metabolic syndrome. The metabolic syndrome (MetS) is composed of the convergence of several cardiometabolic risk factors in a single subject, mainly central obesity, hypertension, insulin resistance, and atherogenic dyslipidemia [1]. Inadequate control of these risk factors can lead to the development of cardiovascular disease. The central adiposity degree during childhood is a strong predictor of obesity, insulin resistance [2], and lipid abnormalities in adulthood [3] and has been significantly associated with the development of cardiovascular risk in young adults [4]. Accordingly, the assessment of central adiposity, among other anthropometric parameters, along with the study of biochemical markers in blood, are essential for the diagnosis of MetS in the adolescent population [5,6].

The exact prevalence of MetS in childhood and adolescence is unknown due to the wide range of physical and hormonal changes associated with age and sex during this stage of life [7]. However, several studies coincide that sedentary behaviors and insufficient physical activity play a key role in the increased prevalence of MetS and related metabolic disturbances among adolescents. Recent evidence has linked higher sedentary time with unfavorable cardiometabolic profiles and an elevated risk of MetS, independent of other lifestyle factors [8,9,10,11]. Consequently, early diagnosis of MetS is considered an important target for implementing interventions that reduce the development of cardiometabolic diseases in later stages of life [12]. Therefore, an inexpensive, reliable, and easily available tool is needed to routinely predict MetS in children and adolescents.

In this context, different investigations have focused on the study of biochemical and anthropometric indices related to lipid profile and visceral adipose function, such as triglyceride glucose (TyG) index [13,14,15,16,17], visceral adiposity index (VAI) [13], logarithm children’s lipid accumulation product (LnCLAP) [14], triglyceride glucose-body mass index (TyG-BMI) [15,16,17], triglyceride glucose-waist circumference (TyG-WC) [15,16,17], and triglyceride glucose-waist-to-hip ratio (TyG-WHR) [18], in order to assess their ability to predict cardiovascular events. Recently, Dundar et al. [13], in their study with Turkish children, found that TyG and VAI show statistically significant cut-off values in both sexes, being effective indexes to predict MetS in children. On the other hand, Rajendran et al. [14], in their study with young adults from India, observed that LnCLAP showed an area under the curve greater than the TyG index in male and female subjects. Therefore, the authors concluded that LnCLAP and the TyG index are significantly associated with MetS in young adults. However, these findings obtained with a young adult population need to be contrasted with an adolescent population. Likewise, TyG-related parameters, such as the product of TyG-BMI, TyG-WC, and TyG-WHR, have demonstrated effectiveness in assessing cardiometabolic disease risk in adult populations [15,16,17,18].

Nevertheless, very few studies have examined the capacity of these indices to predict MetS in adolescent populations. Therefore, we aimed (i) to analyze the capacity of the six biochemical and/or anthropometric indices described to discriminate MetS, (ii) to establish specific cut-off points for these indices, and (iii) to determine which of them offers the highest discriminative accuracy in a Spanish sample of adolescents. Moreover, as many of these indices are influenced by dietary and metabolic factors, their use could be highly relevant in clinical-nutritional settings as part of integrated strategies to promote metabolic health and prevent diet-related chronic conditions. In this context, the present study also contributes to public health by providing evidence-based tools that can support early screening and targeted interventions in adolescents, ultimately helping to reduce the future burden of cardiometabolic diseases.

## 2. Materials and Methods

### 2.1. Study Design and Sample

The results reported in this manuscript are part of a large epidemiological, observational, and cross-sectional study. Based on this study, we have previously published results on the ability to discriminate the presence of metabolic syndrome from different atherogenic indices [19]. However, in this manuscript we address other indices which, although related to the lipid profile and visceral adipose function, are markers of insulin resistance.

The study population comprised 981 adolescents (456 boys and 525 girls), aged 11 to 16 years, enrolled in 18 public and private schools in urban and rural areas of the provinces of Granada and Almeria, in the region of Andalusia, from September 2014 to July 2018. Sample size was calculated for a cross-sectional study with a 95% confidence level (Z = 1.96) and 85% power (beta error = 0.15). Assuming 10% of positive outcomes (MetS), the required total sample size was 874, which was increased to 961 considering 10% losses. The final number of participants was 981 to avoid excluding single participants within schools.

An invitation letter was sent to school principals, and all invited schools agreed to participate. To ensure random selection and reduce selection bias, two classes per grade (out of a total of three) were randomly selected within each school using a computer-generated simple randomization procedure. All students in the selected classes were invited to participate. Eligibility criteria included being in good health and having no diagnosed metabolic disorders or physical limitations. Adolescents who did not meet these criteria were excluded. The study follows the STROBE reporting guidelines [20]. Ethical approval was granted by the Ethics Committee of the University of Granada, and authorization was obtained from each school’s administration. All university protocols were followed, including voluntary participation, confidentiality, anonymity, and informed consent. Written consent was obtained from the parents or legal guardians of all participants, in accordance with the Declaration of Helsinki [21].

### 2.2. Anthropometric Parameters

Anthropometric assessments were conducted following the guidelines of the International Society for the Advancement of Kinanthropometry (ISAK) [22], by the same certified Level 2 ISAK anthropometrist. Measurements were taken between 8:30 and 10:30 a.m., ensuring a 12 h fasting period and 48 h without physical activity prior to evaluation. To ensure participant privacy, all assessments were conducted individually in a private classroom provided by each school.

Body weight (kg) was recorded twice using a self-calibrating Seca 861 Class III digital scale (Saint Paul, MN, USA), accurate to 100 g, with participants barefoot and wearing light clothing [22]. Height was measured using a SECA stadiometer (model 214) with 1 cm precision [22]. Body mass index (BMI) was calculated as weight in kilograms divided by height in meters squared (kg/m^2^) [23].

Waist circumference (WC) was measured using a Seca retractable tape, accurate to 1 mm, at the midpoint between the lowest rib and the iliac crest, at the end of a normal exhalation [22]. Hip circumference (HC) was assessed at the point of greatest gluteal protrusion [22]. The waist-to-hip ratio (WHR) was derived by dividing WC by HC [22]. Skinfold thicknesses (triceps, biceps, subscapular, and suprailiac) were also recorded using a Holtain caliper (Holtain Ltd, Crymych, United Kingdom.), with a measurement accuracy of 0.1–0.2 mm [22].

Body fat percentage was estimated from these skinfolds. First, body density was calculated using the Brook equation [24] and then converted to body fat percentage using the Siri equation [25].

### 2.3. Biochemical Parameters

Venous blood collection was carried out after overnight fasting (12 h) and was performed by qualified professionals. At 8:00 a.m., a 10 mL venous blood sample was collected from the antecubital vein of the right arm using a sterile vacuum collection tube. Within four hours of collection, samples were centrifuged at 1300 g for 15 min (Z400 K; Hermle, Hermle Labortechnik GmbH, Wehingen, Germany). Prior to centrifugation, glucose levels were determined using an enzymatic colorimetric assay kit, following the manufacturer’s instructions [glucose oxidase-phenol aminophenazone (GOD-PAP) method; Human Diagnostics, Wiesbaden, Germany], as well as the concentrations of HDL-cholesterol (HDL-c), total cholesterol, and TG by means of enzymatic colorimetric methods using an Olympus analyzer. LDL-cholesterol (LDL-c) was estimated using the Friedewald equation [(LDL-c) = (total cholesterol) − (HDL-c) − ([TG]/5)], where TG = TG concentration. Serum insulin concentrations were measured using a radioimmunoassay technique (Insulin Kit; DPC, Los Angeles, CA, USA). Insulin resistance was estimated using the Homeostasis Model Assessment (HOMA-IR) [26], calculated with the formula: fasting glucose (mmol/L) × fasting insulin (mU/L)/22.5.

### 2.4. Blood Pressure Determination

Blood pressure (BP) was assessed three times using a calibrated aneroid sphygmomanometer alongside a Littmann^®^ stethoscope, and then the mean of the three measurements was obtained. The procedure followed the American Heart Association’s Subcommittee on Professional and Public Education guidelines for accurate BP assessment [27]. Participants remained seated, silent, and relaxed during the measurement. Systolic BP values ≥ 130 mmHg and/or diastolic BP values ≥ 85 mmHg were considered indicative of elevated cardiometabolic risk.

### 2.5. Metabolic Syndrome Definition

Metabolic syndrome (MetS) was diagnosed based on the International Diabetes Federation (IDF) criteria [28]. According to these standards, MetS in adolescents is confirmed when abdominal obesity (waist circumference ≥ 94 cm in boys and ≥ 80 cm in girls) is present along with at least two of the following risk factors: fasting blood glucose between 100 and 125 mg/dL, triglycerides ≥ 150 mg/dL, HDL-cholesterol < 40 mg/dL in boys or < 50 mg/dL in girls, and/or BP ≥ 130/85 mmHg. Participants meeting these thresholds were classified as having MetS, while those not fulfilling the criteria were categorized as non-MetS.

### 2.6. Biochemical and/or Anthropometric Indices

The biochemical and/or anthropometric indices evaluated in the study were as follows:

- TyG index: The TyG index was calculated as Ln (TG [mg/dL] × glucose [mg/dL]/2) [29].
- Visceral adiposity index (VAI): VAI was estimated using the sex-specific formulas proposed by Amato et al. [30]:
  ○ Males: VAI = (WC (cm)/(39.68 + (1.88 × BMI))) × (TG/1.03) × (1.31/HDL)
  ○ Females: VAI = (WC (cm)/(36.58 + (1.89 × BMI))) × (TG/0.81) × (1.52/HDL)
- LnCLAP index (children’s lipid accumulation product): The CLAP was calculated as waist circumference (WC (cm)) × abdominal skinfold thickness (AST (mm)) × triglyceride (TG (mmol/L))/100 [31].
- TyG-BMI index: The TyG-BMI index was computed according to the equation TyG index × BMI [32]. On the other hand, TyG-WC was calculated by TyG × WC [33]. The TyG-WHR index was obtained by multiplying the TyG index with the WHR [18].

### 2.7. Strategies to Minimize Measurement Bias

To reduce potential measurement bias, standardized procedures were followed across all measurement domains. All anthropometric data were collected by the same ISAK-certified level-2 anthropometrist, following the recommendations of the International Society for the Advancement of Kinanthropometry (ISAK, 2011). Blood pressure was measured according to the standardized guidelines of the American Heart Association, ensuring proper resting conditions and using calibrated equipment. Biochemical parameters were analyzed using validated commercial kits, and all blood samples were processed under strict laboratory protocols. These measures aimed to ensure consistency, accuracy, and reliability in all data collected.

### 2.8. Statistical Analysis

All statistical analyses were conducted using IBM SPSS Statistics v24.0 (IBM Corp., Armonk, NY, USA), with significance set at *p* < 0.05. The Kolmogorov–Smirnov test was used to verify the normality of distribution. Results are expressed as mean ± standard deviation, except for categorical variables (frequencies and percentages). Sex differences in the prevalence of MetS were examined using the chi-square test, and differences in continuous variables were assessed via Student’s *t*-test. Odds ratios between various biochemical and/or anthropometric parameters and MetS were calculated by logistic regression analyses using 3 models. Model 1: non-adjusted model; model 2: adjusted for age; and model 3: adjusted for age, systolic and diastolic blood pressures, body mass index, waist circumference, hip circumference, waist-to-hip ratio, and suprailiac skinfold. Goodness-of-fit was assessed using the Hosmer–Lemeshow test. Receiver operating characteristic (ROC) curve analyses were performed to assess the discriminatory power of each index for MetS, and optimal cut-off values were determined using Youden’s index (sensitivity + specificity − 1).

## 3. Results

### 3.1. Baseline Characteristics of Participants

Table 1 shows baseline biochemical and anthropometric characteristics not reported in our previously published results [19]. 46.48% of the participants were boys, and the rest were girls. Boys exhibited higher values for weight, WC, WHR, TG, and insulin serum levels compared to girls (*p* < 0.05), while girls presented a higher percentage of fat than boys (*p* < 0.05). Mean values of TyG-WC and of TyG-WHR indices were higher in boys than in girls (*p* < 0.001), whereas higher mean values for VAI (*p* < 0.001) and LnCLAP (*p* < 0.05) were observed in girls than in boys.

### 3.2. Logistic Regression Analyses of TyG-Derived Indices, VAI, LnCLAP, and Their Association with MetS

Table 2 shows the findings of the logistic regression models examining the relationship between various TyG-based indices, VAI, and LnCLAP with the presence of MetS in boys. Among all parameters analyzed, the TyG index exhibited the strongest association with MetS, both in the unadjusted model (model 1) and after adjusting by age (model 2). This association was somewhat lower in model 3 after adjustments by age, systolic and diastolic BPs, BMI, WC, HC, WHR, and suprailiac skinfold. In model 3 the strongest association with MetS was observed for the TyG-WHR index, which was the second most strongly associated index with MetS in model 1 and in model 2.

Table 3 presents the logistic regression analyses conducted on female adolescents. In girls, similar results to boys were observed, with the TyG index being the parameter most strongly associated with MetS, with an OR of 7.52 and 7.62 in model 1 and in model 2, respectively. Also, somewhat lower values for the TyG index were observed for model 3, finding in this model that the most strongly associated parameter with MetS was the TyG-BMI index with an OR of 3.68, and the second one the TyG index.

### 3.3. Determination of the Ability of Different Biochemical and/or Anthropometric Indices to Discriminate MetS by Analyzing Receiver Operating Characteristics

Table 4 and Table 5 present the results of the ROC analysis evaluating the discriminative capacity of various indices for detecting MetS in boys and girls, respectively. In both genders, AUC values ranged from 0.84 to 0.94 (*p* < 0.001). Among all indices, VAI yielded the highest AUC in both groups, followed by TyG-WC. The optimal cutoff value for VAI was 1.647 in boys and 2.297 in girls. Regarding TyG-WC, the thresholds were 687.938 for boys and 644.481 for girls.

## 4. Discussion

To address the imperative of mitigating cardiometabolic diseases in adulthood, early identification of MetS during adolescence is essential. Current research seeks novel approaches, such as the investigation of different indices linked to lipid profile and visceral adiposity, which have been applied to predict MetS in adults [14,15,16,17], but with limited exploration in Spanish adolescents [19]. This study aimed to assess the discriminative capacity of six of these indices that are associated with lipid profile and visceral adiposity for MetS in a Spanish adolescent sample.

Our primary findings revealed that, based on the criteria proposed by the IDF, 9.0% of boys and 6.1% of girls in the sample had MetS. Notably, these figures contrast with those reported by Dundar et al. [13], who, in their study on Turkish adolescents, showed not only higher overall prevalence of MetS but also gender differences, since higher prevalence among girls was observed compared to boys (28.2% vs. 14.3%, respectively). This discrepancy may stem from variances in body fat distribution and daily lifestyle behaviors between genders.

In our investigation, boys exhibited higher WC, TG, and insulin serum levels compared to girls, while girls showed higher body fat percentages. These findings differ from Reckziegel et al. [34], who observed higher WC, TG, and insulin serum levels in Brazilian adolescent girls. Such disparities could relate to diverse population characteristics and methodological variances (e.g., age groups, sex distribution, ethnic backgrounds, obesity levels, sample sizes, and reference standards). In addition, sexual dimorphism in body fat accumulation, partly influenced by sex steroid hormones, likely explains the observed differences in body fat percentages between genders [35,36]. Our study also noted higher VAI values among girls, which is aligned with the findings reported by Ejtahed et al. [37] in Iranian adolescents.

Furthermore, our analysis highlighted that TyG-WHR and TyG-BMI were revealed as the most robust MetS indicators for boys and girls, respectively. These results corroborate the findings published by Miao et al. [18] on cardiovascular risk and underscore the potential of these parameters in identifying adolescents at high cardiometabolic risk, aiding in MetS prevention.

In terms of the ability to discriminate between subjects with and without MetS, VAI and TyG-WC emerged as the most effective indices in both genders. The cutoffs observed (1.64 for boys, 2.29 for girls) were consistent with those reported in different international studies [38,39,40]. Variations in cutoffs between genders reflect differences in visceral fat distribution and hormonal influences as suggested elsewhere [35]. These variations have also been reported to be associated with factors such as genetics, gender, age, ethnicity, and puberty [41]. Nevertheless, Rattanatham et al. [42] advocate TyG-WC over VAI for MetS prediction, as they showed superior performance, since they found higher AUC and Youden index values in their ROC analysis for this index (0.892 and 0.620, respectively). Yet, our study achieved higher AUC values and Youden indices in both genders than those reported by Rattanatham et al. [42] for both VAI and TyG-WC (and TyG-BMI for girls), which emphasizes their utility in pediatric research and nutritional health interventions, particularly among adolescents.

The results obtained in our study are relevant to the professional practice of nutritionists and other health professionals, as they reflect the discriminative capacity of biochemical and anthropometric indices. The simplicity and innocuousness of these indices allow their incorporation into diagnostic and follow-up programs for adolescents with MetS. Given their sensitivity to alterations in lipid and glucose metabolism, these markers may also support the nutritional assessment and metabolic monitoring of young populations, facilitating health management and helping to prevent the development of cardiovascular and metabolic disorders, thereby contributing to the reduction in non-communicable diseases.

The study has several strengths, including a substantial, varied cohort of diverse age groups and sexes, thereby bolstering the robustness of the results. Nonetheless, the sample was homogeneous, as it was drawn from a single geographical region with individuals sharing culture, lifestyle, and dietary habits, which also reinforces the validity of the study. Despite its robustness, the study has limitations. For instance, the cross-sectional design, while valuable for capturing data at a specific point in time from a diverse cohort, hindered the ability to establish causal relationships between adiposity measurements and metabolic health. This design limitation prevents us from determining whether adiposity measurements directly influence metabolic health outcomes over time, as it only provides a snapshot of associations at a single time point. Another limitation of the study is the lack of assessment of pubertal development. Longitudinal studies would be necessary to better understand the temporal relationships and potential causality between these variables and should include pubertal staging to more accurately capture developmental differences in adolescent populations.

## 5. Conclusions

Our study shows that the six obesity-related indices assessed are useful in discriminating between adolescents with or without MetS, as is shown in the AUC values obtained in the ROC analyses, especially for the visceral adiposity index and triglyceride glucose-waist circumference index. Logistic regression analyses further revealed that TyG-WHR and TyG-BMI showed the strongest association with MetS in boys and girls, respectively. These findings highlight the potential ability of the studied indices in supporting early identification strategies for adolescents at risk for MetS, a condition closely associated with future cardiovascular disease, one of the leading non-communicable diseases worldwide. However, the results are based on a sample of Spanish adolescents, whose specific lifestyles and habits may influence the findings. Therefore, caution is advised when generalizing to other populations.

Future studies with longitudinal designs are needed to better understand the temporal relationships between these indices and the development of MetS. It would also be important to include adolescents from other populations and to control for pubertal stage in order to confirm the applicability and robustness of these indices in different settings.

## Figures and Tables

**Table 1:** Characteristics of the participants.

Variables	Boys (*n* = 456)	Girls (*n*= 525)	*p*-Value
HC (cm)	84.2 ± 9.9	83.8 ± 8.6	0.510
WHR	0.88 ± 0.06	0.87 ± 0.06	<0.001
TyG	8.5 ± 0.5	8.5 ± 0.4	0.417
VAI	1.7 ± 1.1	2.4 ± 1.7	<0.001
LnCLAP	2.6 ± 0.8	2.7 ± 0.7	0.027
TyG-BMI	183.1 ± 35.8	179.6 ± 32.8	0.113
TyG-WC	629.6 ± 107.7	607.1 ± 89.0	<0.001
TyG-WHR	7.5 ± 0.8	7.2 ± 0.7	<0.001

Data expressed as mean ± SD. BMI, body mass index; CLAP, children’s lipid accumulation product; HC, hip circumference; TyG, triglyceride-glucose; VAI, visceral adiposity index; WC, waist circumference; WHR, waist-to-hip ratio.

**Table 2:** Logistic regression analyses for the relationship between different metabolic/anthropometric indices and MetS in boys.

Variables	Model 1			Model 2			Model 3		
	OR (95% CI)	*p*	GoF	OR (95% CI)	*p*	GoF	OR (95% CI)	*p*	GoF
TyG	5.21 (3.23–8.40)	<0.001	0.039	6.15 (3.73–10.25)	<0.001	0.610	2.78 (1.80–4.29)	<0.001	<0.001
VAI	1.55 (1.27–1.90)	<0.001	<0.001	1.56 (1.28–1.90)	<0.001	<0.001	1.51 (1.23–1.84)	<0.001	<0.001
LnCLAP	1.69 (1.24–2.30)	<0.001	<0.001	1.72 (1.26–2.35)	<0.001	<0.001	3.14 (1.57–6.30)	0.001	<0.001
TyG-BMI	1.01 (1.01–1.02)	<0.001	<0.001	1.01 (1.00–1.02)	<0.001	<0.001	1.06 (1.01–1.10)	<0.001	<0.001
TyG-WC	1.00 (1.00–1.01)	<0.001	<0.001	1.01 (1.00–1.01)	<0.001	<0.001	1.02 (1.01–1.02)	<0.001	<0.001
TyG-WHR	2.02 (1.46–2.79)	<0.001	<0.001	2.06 (1.49–2.86)	<0.001	<0.001	3.19 (2.96–5.20)	<0.001	<0.001
Glucose	1.02 (1.01–1.02)	<0.001	<0.001	1.02 (1.01–1.02)	<0.001	<0.001	1.02 (1.01–1.02)	<0.001	<0.001
TG	1.01 (1.00–1.01)	<0.001	<0.001	1.01 (1.00–1.02)	<0.001	0.068	1.01 (1.00–1.01)	<0.001	<0.001
Cholesterol	1.55 (1.26–1.91)	<0.001	<0.001	1.55 (1.26–1.92)	<0.001	<0.001	1.51 (1.22–1.87)	<0.001	<0.001
HDL-c	0.82 (0.76–0.88)	<0.001	<0.001	0.82 (0.76–0.88)	<0.001	<0.001	0.83 (0.77–0.89)	<0.001	<0.001

Model 1: non-adjusted model; Model 2: adjusted for age; Model 3: adjusted for age, systolic and diastolic blood pressures, body mass index, waist circumference, hip circumference, waist-to-hip ratio, and suprailiac skinfold. BMI, body mass index; CLAP, children’s lipid accumulation product; CI, confidence interval; GoF, goodness of fit; HDL-c, high-density lipoprotein cholesterol; MetS, metabolic syndrome; OR, odds ratio; TG, triglycerides; TyG, triglyceride-glucose index; VAI, visceral adiposity index; WC, waist circumference; WHR, waist-to-hip ratio. Model fit was assessed using the Hosmer–Lemeshow GoF test.

**Table 3:** Logistic regression analyses for the relationship between different metabolic/anthropometric indices and MetS in girls.

Variables	Model 1			Model 2			Model 3		
	OR (95% CI)	*p*	GoF	OR (95% CI)	*p*	GoF	OR (95% CI)	*p*	GoF
TyG	7.52 (4.29–13.20)	<0.001	0.026	7.62 (4.35–13.36)	<0.001	<0.001	2.87 (1.79–4.61)	<0.001	<0.001
VAI	1.19 (1.03–1.38)	0.021	<0.001	1.19 (1.03–1.38)	0.021	<0.001	1.18 (1.02–1.36)	0.022	<0.001
LnCLAP	1.03 (1.02–1.04)	<0.001	<0.001	1.03 (1.02–1.04)	<0.001	<0.001	1.04 (1.02–1.07)	0.001	<0.001
TyG-BMI	1.71 (1.18–2.46)	0.004	<0.001	1.71 (1.19–2.47)	0.004	<0.001	3.68 (1.57–8.65)	0.003	<0.001
TyG-WC	1.01 (1.00–1.02)	<0.001	<0.001	1.01 (1.01–1.02)	<0.001	<0.001	1.05 (1.02–1.10)	<0.001	<0.001
TyG-WHR	1.01 (1.00–1.01)	<0.001	<0.001	1.01 (1.00–1.01)	<0.001	<0.001	1.02 (1.01–1.02)	<0.001	<0.001
Glucose	1.01 (1.01–1.02)	<0.001	<0.001	1.01 (1.01–1.02)	<0.001	<0.001	1.01 (1.01–1.02)	<0.001	<0.001
TG	1.01 (1.01–1.01)	<0.001	<0.001	1.01 (1.01–1.02)	<0.001	0.017	1.01 (1.00–1.01)	<0.001	<0.001
Cholesterol	1.56 (1.25–1.95)	<0.001	<0.001	1.56 (1.25–1.95)	<0.001	<0.001	1.57 (1.25–1.7)	<0.001	<0.001
HDL-c	0.82 (0.76–0.88)	<0.001	<0.001	0.82 (0.76–0.88)	<0.001	<0.001	0.83 (0.77–0.89)	<0.001	<0.001

Model 1: non-adjusted model; Model 2: adjusted for age; Model 3: adjusted for age, systolic and diastolic blood pressures, body mass index, waist circumference, hip circumference, waist-to-hip ratio, and suprailiac skinfold. BMI, body mass index; CLAP, children’s lipid accumulation product; CI, confidence interval; GoF, goodness of fit; HDL-c, high-density lipoprotein cholesterol; MetS, metabolic syndrome; OR, odds ratio; TG, triglycerides; TyG, triglyceride-glucose index; VAI, visceral adiposity index; WC, waist circumference; WHR, waist-to-hip ratio. Model fit was assessed using the Hosmer–Lemeshow GoF test.

**Table 4:** AUC, optimal cutoff value, sensitivity, specificity, and Youden index from the ROC analysis of biochemical and/or anthropometric indices for identifying MetS in boys.

Variables	AUC ± SD	95% CI	*p*	Cutoff	Sensitivity	Specificity	Youden Index
TyG	0.847 ± 0.039	0.771–0.922	<0.001	8.534	0.829	0.814	0.644
VAI	0.926 ± 0.016	0.894–0.957	<0.001	1.647	0.854	0.862	0.716
LnCLAP	0.847 ± 0.026	0.796–0.898	<0.001	3.228	0.756	0.769	0.524
TyG-BMI	0.874 ± 0.023	0.829–0.919	<0.001	201.549	0.780	0.810	0.590
TyG-WC	0.908 ± 0.018	0.874–0.943	<0.001	687.938	0.878	0.824	0.702
TyG-WHR	0.881 ± 0.022	0.37–0.925	<0.001	7.706	0.780	0.778	0.559

AUC, area under the curve; BMI, body mass index; CLAP, children’s lipid accumulation product; CI, confidence interval; MetS, metabolic syndrome; ROC, receiver operator characteristic; SD, standard deviation; TyG, triglyceride-glucose index; VAI, visceral adiposity index; WC, waist circumference; WHR, waist-to-hip ratio. The Youden index was calculated as sensitivity + specificity − 1.

**Table 5:** AUC, optimal cutoff value, sensitivity, specificity, and Youden index from the ROC analysis of biochemical and/or anthropometric indices for identifying MetS in girls.

Variables	AUC ± SD	95% CI	*p*	Cutoff	Sensitivity	Specificity	Youden Index
TyG	0.896 ± 0.037	0.823–0.968	<0.001	8.567	0.938	0.850	0.787
VAI	0.935 ± 0.021	0.894–0.977	<0.001	2.297	0.969	0.793	0.762
LnCLAP	0.846 ± 0.034	0.780–0.912	<0.001	3.033	0.844	0.686	0.529
TyG-BMI	0.918 ± 0.019	0.881–0.954	<0.001	210.932	0.781	0.895	0.676
TyG-WC	0.927 ± 0.016	0.896–0.958	<0.001	644.481	0.969	0.769	0.738
TyG-WHR	0.867 ± 0.037	0.793–0.940	<0.001	7.493	0.875	0.799	0.674

AUC, area under the curve; BMI, body mass index; CLAP, children’s lipid accumulation product; CI, confidence interval; MetS, metabolic syndrome; ROC, receiver operator characteristic; SD, standard deviation; TyG, triglyceride-glucose index; VAI, visceral adiposity index; WC, waist circumference; WHR, waist-to-hip ratio. The Youden index was calculated as sensitivity + specificity − 1.

## Data Availability

The data presented in this study are available on request from the corresponding author. The data are not publicly available due to the need to maintain the privacy of participants.

## Abbreviations

The following abbreviations are used in this manuscript:

BP	Blood pressure
BMI	Body mass index
GOD-PAP	Glucose oxidase-phenol aminophenazone
HDL-c	HDL-cholesterol
HC	Hip circumference
HOMA-IR	Homeostasis Model Assessment-Insulin Resistance
ISAK	International Society for the Advancement of Kinanthropometry
LnCLAP	Logarithm children’s lipid accumulation product
LDL-c	LDL-cholesterol
MetS	Metabolic syndrome
TG	Triglycerides
TyG	Triglyceride glucose
TyG-BMI	Triglyceride glucose-body mass index
TyG-WC	Triglyceride glucose-waist circumference
TyG-WHR	Triglyceride glucose-waist-to-hip ratio
VAI	Visceral adiposity index
WC	Waist circumference
WHR	Waist-to-hip ratio

## References

[1] Codazzi V., Frontino G., Galimberti L., Giustina A., Petrelli A. (2023). Mechanisms and Risk Factors of Metabolic Syndrome in Children and Adolescents. Endocrine.

[2] Steinberger J., Moran A., Hong C.-P., Jacobs D.R., Sinaiko A.R. (2001). Adiposity in Childhood Predicts Obesity and Insulin Resistance in Young Adulthood. J. Pediatr..

[3] Viitasalo A., Pitkänen N., Pahkala K., Lehtimäki T., Viikari J.S.A., Raitakari O., Kilpeläinen T.O. (2020). Increase in Adiposity from Childhood to Adulthood Predicts a Metabolically Obese Phenotype in Normal-Weight Adults. Int. J. Obes..

[4] Morrison J.A., Friedman L.A., Wang P., Glueck C.J. (2008). Metabolic Syndrome in Childhood Predicts Adult Metabolic Syndrome and Type 2 Diabetes Mellitus 25 to 30 Years Later. J. Pediatr..

[5] Konuthula D., Tan M.M., Burnet D.L. (2023). Challenges and Opportunities in Diagnosis and Management of Cardiometabolic Risk in Adolescents. Curr. Diab. Rep..

[6] Lischka: J., Schanzer A., De Gier C., Greber-Platzer S., Zeyda M. (2023). Macrophage-Associated Markers of Metaflammation Are Linked to Metabolic Dysfunction in Pediatric Obesity. Cytokine.

[7] Kim J.W., Park S.H., Kim Y., Im M., Han H.-S. (2016). The Cutoff Values of Indirect Indices for Measuring Insulin Resistance for Metabolic Syndrome in Korean Children and Adolescents. Ann. Pediatr. Endocrinol. Metab..

[8] Aravindakshan S.S., David A., Saradakutty G., Agarwal P. (2024). Prevalence of Metabolic Syndrome and Its Associated Risk Factors Among Schoolchildren Aged 11-13 Years Living in Thiruvananthapuram District, Kerala, India: A Nested Case-Control Study. Cureus.

[9] Mendonça G., Barbosa A.O., Moura I.R.D., Silva J.M.D.P.F., Prazeres Filho A., da Silva D.J., Toscano C.V.A., de Farias Júnior J.C. (2025). Sedentary Behavior and Cardiometabolic Markers in Adolescents: A 4-Year Longitudinal Study. Pediatr. Exerc. Sci..

[10] Cheng X., Guo Q., Ju L., Gong W., Wei X., Xu X., Zhao L., Fang H. (2024). Association between Sedentary Behavior, Screen Time and Metabolic Syndrome among Chinese Children and Adolescents. BMC Public Health.

[11] Andrade C. (2024). Physical Exercise and Health, 5: Sedentary Time, Independent of Health-Related Physical Activity, as a Risk Factor for Adverse Physical Health and Mental Health Outcomes. J. Clin. Psychiatry.

[12] Leis R., De Lamas C., De Castro M.-J., Picáns R., Gil-Campos M., Couce M.L. (2019). Effects of Nutritional Education Interventions on Metabolic Risk in Children and Adolescents: A Systematic Review of Controlled Trials. Nutrients.

[13] Dundar C., Terzi O., Arslan H.N. (2023). Comparison of the Ability of HOMA-IR, VAI, and TyG Indexes to Predict Metabolic Syndrome in Children with Obesity: A Cross-Sectional Study. BMC Pediatr..

[14] Rajendran S., Kizhakkayil Padikkal A.K., Mishra S., Madhavanpillai M. (2022). Association of Lipid Accumulation Product and Triglyceride-Glucose Index with Metabolic Syndrome in Young Adults: A Cross-Sectional Study. Int. J. Endocrinol. Metab..

[15] Raimi T.H., Dele-Ojo B.F., Dada S.A., Fadare J.O., Ajayi D.D., Ajayi E.A., Ajayi O.A. (2021). Triglyceride-Glucose Index and Related Parameters Predicted Metabolic Syndrome in Nigerians. Metab. Syndr. Relat. Disord..

[16] Gui J., Li Y., Liu H., Guo L., Li J., Lei Y., Li X., Sun L., Yang L., Yuan T. (2023). Obesity- and Lipid-Related Indices as a Predictor of Obesity Metabolic Syndrome in a National Cohort Study. Front. Public Health.

[17] Lim J., Kim J., Koo S.H., Kwon G.C. (2019). Comparison of Triglyceride Glucose Index, and Related Parameters to Predict Insulin Resistance in Korean Adults: An Analysis of the 2007–2010 Korean National Health and Nutrition Examination Survey. PLoS ONE.

[18] Miao H., Zhou Z., Yang S., Zhang Y. (2024). The Association of Triglyceride-Glucose Index and Related Parameters with Hypertension and Cardiovascular Risk: A Cross-Sectional Study. Hypertens. Res..

[19] Fernández-Aparicio Á., Perona J.S., Schmidt-RioValle J., Padez C., González-Jiménez E. (2022). Assessment of Different Atherogenic Indices as Predictors of Metabolic Syndrome in Spanish Adolescents. Biol. Res. Nurs..

[20] von Elm E., Altman D.G., Egger M., Pocock S.J., Gøtzsche P.C., Vandenbroucke J.P. (2008). STROBE Initiative The Strengthening the Reporting of Observational Studies in Epidemiology (STROBE) Statement: Guidelines for Reporting Observational Studies. J. Clin. Epidemiol..

[21] World Medical Association Declaration of Helsinki (1997). Recommendations Guiding Physicians in Biomedical Research Involving Human Subjects. JAMA.

[22] Stewart A., Marfell-Jones M., Olds T., De Ridder H. (2011). International Standards for Anthropometric Assessment.

[23] World Health Organization Obesity and Overweight. https://www.who.int/news-room/fact-sheets/detail/obesity-and-overweight.

[24] Brook C.G.D. (1971). Determination of Body Composition of Children from Skinfold Measurements. Arch. Dis. Child..

[25] Siri W.E. (1993). Body Composition from Fluid Spaces and Density: Analysis of Methods. 1961. Nutrition.

[26] Matthews D.R., Hosker J.P., Rudenski A.S., Naylor B.A., Treacher D.F., Turner R.C. (1985). Homeostasis Model Assessment: Insulin Resistance and β-Cell Function from Fasting Plasma Glucose and Insulin Concentrations in Man. Diabetologia.

[27] Pickering T.G., Hall J.E., Appel L.J., Falkner B.E., Graves J., Hill M.N., Jones D.W., Kurtz T., Sheps S.G., Roccella E.J. (2005). Recommendations for Blood Pressure Measurement in Humans and Experimental Animals: Part 1: Blood Pressure Measurement in Humans: A Statement for Professionals From the Subcommittee of Professional and Public Education of the American Heart Association Council on High Blood Pressure Research. Circulation.

[28] Zimmet P., Alberti K.G.M., Kaufman F., Tajima N., Silink M., Arslanian S., Wong G., Bennett P., Shaw J., Caprio S. (2007). The Metabolic Syndrome in Children and Adolescents—an IDF Consensus Report. Pediatr. Diabetes.

[29] Guerrero-Romero F., Simental-Mendía L.E., González-Ortiz M., Martínez-Abundis E., Ramos-Zavala M.G., Hernández-González S.O., Jacques-Camarena O., Rodríguez-Morán M. (2010). The Product of Triglycerides and Glucose, a Simple Measure of Insulin Sensitivity. Comparison with the Euglycemic-Hyperinsulinemic Clamp. J. Clin. Endocrinol. Metab..

[30] Amato M.C., Giordano C., Galia M., Criscimanna A., Vitabile S., Midiri M., Galluzzo A., AlkaMeSy Study Group Visceral Adiposity Index (2010). A Reliable Indicator of Visceral Fat Function Associated with Cardiometabolic Risk. Diabetes Care.

[31] Zhang Y., Hu J., Li Z., Li T., Chen M., Wu L., Liu W., Han H., Yao R., Fu L. (2019). A Novel Indicator Of Lipid Accumulation Product Associated With Metabolic Syndrome In Chinese Children And Adolescents. DMSO.

[32] Er L.-K., Wu S., Chou H.-H., Hsu L.-A., Teng M.-S., Sun Y.-C., Ko Y.-L. (2016). Triglyceride Glucose-Body Mass Index Is a Simple and Clinically Useful Surrogate Marker for Insulin Resistance in Nondiabetic Individuals. PLoS ONE.

[33] Li S., Feng L., Ding J., Zhou W., Yuan T., Mao J. (2023). Triglyceride Glucose-Waist Circumference: The Optimum Index to Screen Nonalcoholic Fatty Liver Disease in Non-Obese Adults. BMC Gastroenterol.

[34] Reckziegel M.B., Nepomuceno P., Machado T., Renner J.D.P., Pohl H.H., Nogueira-de-Almeida C.A., Mello E.D.D. (2023). The Triglyceride-Glucose Index as an Indicator of Insulin Resistance and Cardiometabolic Risk in Brazilian Adolescents. Arch. Endocrinol. Metab..

[35] Bredella M.A., Mauvais-Jarvis F. (2017). Sex Differences in Body Composition. Sex and Gender Factors Affecting Metabolic Homeostasis, Diabetes and Obesity.

[36] Wells J.C.K. (2007). Sexual Dimorphism of Body Composition. Best Pract. Res. Clin. Endocrinol. Metab..

[37] Ejtahed H.-S., Kelishadi R., Hasani-Ranjbar S., Angoorani P., Motlagh M.E., Shafiee G., Ziaodini H., Taheri M., Qorbani M., Heshmat R. (2019). Discriminatory Ability of Visceral Adiposity Index as an Indicator for Modeling Cardio-Metabolic Risk Factors in Pediatric Population: The CASPIAN-V Study. J. Cardiovasc. Thorac. Res..

[38] Dikaiakou E., Vlachopapadopoulou E.A., Paschou S.A., Athanasouli F., Panagiotopoulos Ι., Kafetzi M., Fotinou A., Michalacos S. (2020). Τriglycerides-Glucose (TyG) Index Is a Sensitive Marker of Insulin Resistance in Greek Children and Adolescents. Endocrine.

[39] Dong Y., Bai L., Cai R., Zhou J., Ding W. (2021). Visceral Adiposity Index Performed Better than Traditional Adiposity Indicators in Predicting Unhealthy Metabolic Phenotype among Chinese Children and Adolescents. Sci. Rep..

[40] Vizzuso S., Del Torto A., Dilillo D., Calcaterra V., Di Profio E., Leone A., Gilardini L., Bertoli S., Battezzati A., Zuccotti G.V. (2021). Visceral Adiposity Index (VAI) in Children and Adolescents with Obesity: No Association with Daily Energy Intake but Promising Tool to Identify Metabolic Syndrome (MetS). Nutrients.

[41] Negrea M.O., Neamtu B., Dobrotă I., Sofariu C.R., Crisan R.M., Ciprian B.I., Domnariu C.D., Teodoru M. (2021). Causative Mechanisms of Childhood and Adolescent Obesity Leading to Adult Cardiometabolic Disease: A Literature Review. Appl. Sci..

[42] Rattanatham R., Tangpong J., Chatatikun M., Sun D., Kawakami F., Imai M., Klangbud W.K. (2023). Assessment of Eight Insulin Resistance Surrogate Indexes for Predicting Metabolic Syndrome and Hypertension in Thai Law Enforcement Officers. PeerJ.

